# Supplementary material for: The Systems Biology Research Tool: evolvable open-source software
Source: BMC Syst Biol. 2008 Jun 29;2:55. doi: 10.1186/1752-0509-2-55 (PMC2446383; doi:10.1186/1752-0509-2-55)
Supplement: Additional file 1 — SBRT Archive. An archive of the current version of the Systems Biology Research Tool. [file 1752-0509-2-55-S1.zip › sbrt-1.4.0/doc/developers_guide/api/sbrt/kernel/fba/proc/FluxActivityProcess.html]

FluxActivityProcess


|  |  |  |
| --- | --- | --- |
| **Overview** \| **Package** \| **Class** \| **Use** \| **Tree** \| **Deprecated** \| **Index** \| **Help** | |  |
| **PREV CLASS**   **NEXT CLASS** | **FRAMES**    **NO FRAMES**     **All Classes** |  |
| SUMMARY: NESTED \| FIELD \| CONSTR \| METHOD | DETAIL: FIELD \| CONSTR \| METHOD |  |


---


## sbrt.kernel.fba.proc Class FluxActivityProcess

```
java.lang.Object
  sbrt.kernel.fba.proc.FbaProcess<FluxActivityProcess.Results>
      sbrt.kernel.fba.proc.FluxActivityProcess
```

**All Implemented Interfaces:**: KernelProcess<FluxActivityProcess.Results>, MonitoredProcess<FluxActivityProcess.Results>

---

``` public final class FluxActivityProcess extends FbaProcess<FluxActivityProcess.Results> implements MonitoredProcess<FluxActivityProcess.Results> ```

This class is used to perform flux activity experiments.

**Author:**
:   This class was written and documented by Jeremiah Wright
    while in the Wagner lab.

---

| **Nested Class Summary** | |
| --- | --- |
| `static class` | `FluxActivityProcess.Results`             This class is used to represent the results of a flux activity experiment. |


**Constructor Summary**

`FluxActivityProcess(Fluxome fluxome)`
  
          Constructs a new flux activity experiment.


| **Method Summary** | |
| --- | --- |
| `Fluxome` | `getFluxome()`             Returns the fluxome used in this flux activity experiment. |
| `ProgressMonitor` | `getProgress()`             Returns a progress monitor for this experiment. |
| `void` | `run(ResultsManager<FluxActivityProcess.Results> resultsManager)`             Iterates thru the flux vector source set via `setFluxVectorSource(Iterable)`, counts the number of active and inactive fluxes in each vector, and adds the results of each to the provided results manager. |
| `void` | `setFluxVectorSource(java.lang.Iterable<java.util.Map<java.lang.String,java.lang.Double>> vectorSource)`             Sets the flux vector source. |
| `void` | `setZeroCutoff(double zeroCutoff)`             Sets the zero cutoff to the provided value. |

**Methods inherited from class sbrt.kernel.fba.proc.FbaProcess**

`toString`

**Methods inherited from class java.lang.Object**

`clone, equals, finalize, getClass, hashCode, notify, notifyAll, wait, wait, wait`

**Constructor Detail**

### FluxActivityProcess

```
public FluxActivityProcess(Fluxome fluxome)
```

:   Constructs a new flux activity experiment.

    **Parameters:**: `fluxome` - the fluxome for which flux activities will be determined.


**Method Detail**

### getFluxome

```
public Fluxome getFluxome()
```

:   Returns the fluxome used in this flux activity experiment.

    :   **Specified by:**: `getFluxome` in class `FbaProcess<FluxActivityProcess.Results>`
    :   **Returns:**: the fluxome used in this flux activity experiment.

---


### getProgress

```
public ProgressMonitor getProgress()
```

:   Returns a progress monitor for this experiment.

    :   **Specified by:**: `getProgress` in interface `MonitoredProcess<FluxActivityProcess.Results>`
    :   **Returns:**: a progress monitor for this experiment.

---


### setFluxVectorSource

```
public void setFluxVectorSource(java.lang.Iterable<java.util.Map<java.lang.String,java.lang.Double>> vectorSource)
```

:   Sets the flux vector source. These are the flux vectors for which flux
    activities will be measured.

    :   **Parameters:**: `vectorSource` - a source of reaction names mapped to their corresponding flux values.

---


### setZeroCutoff

```
public void setZeroCutoff(double zeroCutoff)
```

:   Sets the zero cutoff to the provided value. The zero cutoff is the value
    used to decide which computed values are close enough to zero to be
    considered equal to zero. This value is an upper bound and should itself
    be close to zero, like 1E-6 for example.

    :   **Parameters:**: `zeroCutoff` - the zero cutoff.

---


### run

```
public void run(ResultsManager<FluxActivityProcess.Results> resultsManager)
```

:   Iterates thru the flux vector source set via
    `setFluxVectorSource(Iterable)`, counts the number of active and
    inactive fluxes in each vector, and adds the results of each to the
    provided results manager.

    :   **Specified by:**: `run` in interface `KernelProcess<FluxActivityProcess.Results>`
    :   **Parameters:**: `resultsManager` - the results manager to which each set of results is added.


---


|  |  |  |
| --- | --- | --- |
| **Overview** \| **Package** \| **Class** \| **Use** \| **Tree** \| **Deprecated** \| **Index** \| **Help** | |  |
| **PREV CLASS**   **NEXT CLASS** | **FRAMES**    **NO FRAMES**     **All Classes** |  |
| SUMMARY: NESTED \| FIELD \| CONSTR \| METHOD | DETAIL: FIELD \| CONSTR \| METHOD |  |


---
